# Efficacy and safety of ferric derisomaltose (FDI) compared with iron sucrose (IS) in patients with iron deficiency anemia after bariatric surgery

**DOI:** 10.1007/s11695-021-05858-0

**Published:** 2022-01-08

**Authors:** Michael Auerbach, Maureen M. Achebe, Lars L. Thomsen, Richard J. Derman

**Affiliations:** 1 Department of Medicine, Georgetown University School of Medicine, Washington, DC USA; 2 Brigham and Women’s Hospital, Dana Farber Cancer Institute, Harvard Medical School, Boston, MA USA; 3 Department of Clinical and Non-Clinical Research, Pharmacosmos A/S, Holbæk, Denmark; 4 Thomas Jefferson University, Philadelphia, PA USA

**Keywords:** Iron deficiency anemia, Ferric derisomaltose, Iron isomaltoside, Iron sucrose, Bariatric surgery

## Abstract

**Purpose:**

Iron deficiency is common following bariatric surgery, and treatment with intravenous iron is often required. This post hoc analysis of data from two randomized, open-label, multicenter trials evaluated the efficacy and safety of ferric derisomaltose (FDI; formerly iron isomaltoside 1000) versus iron sucrose (IS) over 4 weeks in adults with iron deficiency anemia (IDA) resulting from prior bariatric surgery.

**Materials and methods:**

Data were pooled for participants who received FDI or IS in the PROVIDE or FERWON-IDA trials for the treatment of IDA post bariatric surgery. Efficacy outcomes included changes in hemoglobin (Hb) and iron parameters; safety outcomes included the incidence of adverse drug reactions (ADRs), serious or severe hypersensitivity reactions (HSRs), and hypophosphatemia.

**Results:**

The analysis included 159 patients. Mean (standard deviation) cumulative iron doses were 1199 (± 347) mg for FDI and 937 (± 209) mg for IS. Compared with IS, FDI resulted in a faster and more pronounced Hb response, and a higher proportion of responders (Hb level increase ≥ 2 g/dL from baseline) at all time points. The incidence of ADRs was similar with FDI and IS (15.1% and 18.2%, respectively), with no serious ADRs or serious or severe HSRs reported. The incidence of hypophosphatemia was low and similar in both treatment groups, with no cases of severe hypophosphatemia observed.

**Conclusions:**

In patients with IDA resulting from bariatric surgery, FDI produced a faster and more pronounced Hb response than IS. Both FDI and IS were well tolerated.

**Graphical abstract:**

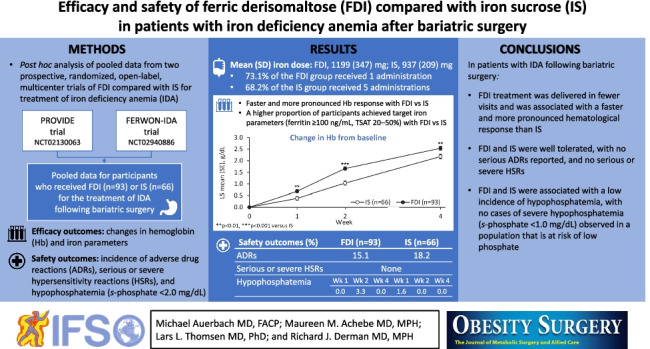

## Introduction

Iron deficiency (ID) is a common cause of anemia following bariatric surgery [1, 2], especially after Roux-en-Y gastric bypass (RYGB), which restricts food intake and nutrient absorption [3–5]. Indeed, Gesquiere et al. reported a 37.2% incidence of ID within 5 years of RYGB [6].

Multiple factors are associated with the development of ID post bariatric surgery, including a reduction in the surface area available for iron absorption (due to decreased stomach capacity and bypass of the duodenum), an inadequate intake of dietary iron (due to a low tolerance of red meat), and reduced gastric acid secretion [2, 4, 7, 8]. Gastric acid is required for the conjugation of iron to vitamin C, amino acids, and sugar, which prevent its conversion to unabsorbable ferric hydroxide in the proximal duodenum [9].

In addition, ID is frequently observed in individuals with obesity [10, 11], which may be due to adiposity-related chronic inflammation inhibiting the absorption of iron [12, 13]. Pre-existing ID can be exacerbated following bariatric surgery [10, 11]. ID can have a considerable impact on an individual’s health, particularly when the deficiency results in anemia. Anemia can manifest as fatigue, dizziness, and shortness of breath; in severe cases, anemia can lead to neurological damage and even heart failure [14]. Other symptoms include pagophagia (a pathological craving for ice) [15], which can impair dentition, and restless legs syndrome, which interferes with sleep resulting in fatigue and impaired quality of life [9, 16]. Therefore, treating ID and any associated anemia is essential.

Clinical practice guidelines recommend that ID following bariatric surgery be treated with oral iron or intravenous (IV) iron [17, 18]. Collaborative guidelines recommend that high doses of oral iron should be the first-line treatment for ID/iron deficiency anemia (IDA) with IV iron reserved only for individuals with severe intolerance to oral iron or with treatment-refractory ID/IDA [18]. Increasing evidence suggests that these guidelines need revisiting. Daily, high-dose oral iron increases hepcidin levels, which in turn reduces iron absorption [19]. Indeed, studies have reported increased iron absorption with alternate-day versus once- or twice-daily dosing [20, 21], suggesting that daily oral iron is unnecessary and even counterproductive.

European guidelines imply that IV iron supplementation can be administered to correct ID without an initial trial of oral iron [17]. Evidence suggests that IV iron may be preferable to oral iron for the treatment of ID/IDA following bariatric surgery. Clinical studies have shown a decline in the ability of individuals to absorb oral iron in the months following bariatric surgery [2, 6, 22]. Additionally, significant gastrointestinal side effects are often reported with oral iron, which can lead to poor treatment adherence [5, 23]. Compared with oral iron, IV iron treatment has been shown to result in fewer adverse events in patients with ID post bariatric surgery, and in faster normalization of iron parameters and a lower reoccurrence of ID in the 12 months following iron supplementation [24]. Furthermore, the IV route avoids exacerbating the existing gastrointestinal perturbations, which are present in individuals whose gastrointestinal tracts have been rerouted [9].

Newer IV iron formulations are approved for administration in high single doses, which minimize the number of infusions needed for iron repletion and the likelihood of requiring retreatment [25–31]. Consequently, high-dose formulations result in fewer visits than low-dose formulations, thereby reducing costs [25, 27, 28], while increasing convenience for patients and practitioners.

Despite the advantages, there is a degree of reluctance to use IV iron due to the perception that it can cause severe hypersensitivity reactions (HSRs) [9]. In reality, although infusion reactions can occur with all IV iron products, the majority of reactions are minor and easily managed, and serious or severe HSRs are rare [32–34].

Hypophosphatemia is a concern with some IV iron formulations [35–38]. While clinical sequelae are uncommon, especially after only one or two doses, persistent hypophosphatemia after multiple doses (often needed post bariatric surgery) can lead to short- and long-term clinical consequences such as fatigue, muscle weakness, osteomalacia, bone pain, and fractures [35, 36, 38].

Ferric derisomaltose (FDI) (formerly known as iron isomaltoside 1000) is a high-dose IV iron formulation [39, 40], which has shown good efficacy and safety in clinical trials for the treatment of ID/IDA across various specialties, including gastroenterology [41, 42]. Two such clinical trials—PROVIDE and FERWON-IDA—compared the efficacy and safety of FDI with that of iron sucrose (IS; a low-dose IV iron formulation) in patients with IDA of various etiologies, including prior bariatric surgery [43–46]. In the PROVIDE and FERWON-IDA trials, FDI demonstrated a more rapid improvement in hematological parameters than IS, with similar low rates of serious or severe HSRs, serious adverse drug reactions (ADRs), and hypophosphatemia [43, 44]. Although the hematological response in FERWON-IDA was, initially, faster with FDI versus IS, the change in hemoglobin (Hb) at the end of the 8-week follow-up period was similar in both treatment groups [44].

The present post hoc analysis was performed on pooled data from the PROVIDE and FERWON-IDA trials to evaluate the efficacy and safety of FDI compared with IS in patients with IDA resulting from prior bariatric surgery.

## Materials and methods

### Trial design

This was a post hoc analysis of pooled data from two prospective, randomized, open-label, comparative, multicenter trials, which evaluated the efficacy and safety of FDI compared with IS in the treatment of IDA: PROVIDE (NCT02130063) and FERWON-IDA (NCT02940886) [43, 44]. This analysis was conducted in the trial participants with IDA resulting from prior bariatric surgery (gastric bypass, gastric banding, obesity surgery, metabolic surgery, or gastrectomy [sleeve gastrectomy]) (Table 1). The designs of these two trials have been described previously [43, 44]. This pooled analysis was not pre-specified in the protocols of the two trials.

**Table 1:** Trials included in the pooled analysis

	PROVIDE	FERWON-IDA
Analysis population	IDA resulting from prior bariatric surgery (gastric bypass, obesity surgery, or gastrectomy [sleeve gastrectomy])	IDA resulting from prior bariatric surgery (gastric banding, gastric bypass, metabolic surgery, or gastrectomy [sleeve gastrectomy])
IDA criteria	Hb < 11.0 g/dL, TSAT < 20%, and *s*-ferritin < 100 ng/mL	Hb ≤ 11.0 g/dL, TSAT < 20%, and *s*-ferritin < 100 ng/mL
Patient numbers	FDI: N = 27	FDI: N = 66
	IS: N = 19	IS: N = 47
IV iron dosing	FDI: cumulative dose of 1000 mg, 1500 mg, or 2000 mg depending on Hb level and body weight	FDI: single dose of 1000 mg
	IS: cumulative dose according to label and calculated using the Ganzoni formula (maximum cumulative dose: 2000 mg)	IS: cumulative dose according to label (recommended cumulative dose: 1000 mg)
Trial duration	5 weeks	10–15 weeks
Reference	Derman R, et al. Am J Hematol 2017; 92(3):286–91	Auerbach M, et al. Am J Hematol 2019; 94(9):1007–14

FDI, ferric derisomaltose/iron isomaltoside 1000; Hb, hemoglobin; IDA, iron deficiency anemia; IS, iron sucrose; IV, intravenous; N, number of patients; *s*-ferritin, serum ferritin; TSAT, transferrin saturation

### Participants

The trials were conducted at 123 sites in the USA; the 74 sites that treated post bariatric surgery patients were included in this pooled analysis. Adults ≥ 18 years of age with IDA of various etiologies, and with a documented history of intolerance or a lack of response to oral iron, or with a clinical need for rapid repletion of iron stores, were eligible for enrolment. IDA was defined as an Hb concentration < 11.0 g/dL (in PROVIDE) or ≤ 11.0 g/dL (in FERWON-IDA), transferrin saturation (TSAT) < 20%, and serum ferritin (*s*-ferritin) < 100 ng/mL (Table 1). The full list of inclusion and exclusion criteria is presented in the publication for each trial [43, 44].

### Interventions

Participants were randomized 2:1 to receive treatment with FDI (Monofer^®^/Monoferric^®^, Pharmacosmos A/S, Holbæk, Denmark [39, 40]) or IS (Venofer^®^, American Regent, Shirley, New York, USA [45, 46]). In the PROVIDE trial, FDI was administered weekly as 1000 mg IV infusions or 500 mg bolus injections, to achieve a cumulative dose of 1000 mg, 1500 mg, or 2000 mg depending on Hb concentration and body weight. IS was administered as 200 mg IV infusions up to two times per week to achieve a cumulative dose calculated using the Ganzoni formula; the maximum cumulative dose of IS was 2000 mg. In the FERWON-IDA trial, FDI was administered as a single 1000 mg IV infusion at baseline. IS was administered as 200 mg IV injections, which were repeated up to five times. The recommended cumulative dose of IS was 1000 mg. During the trials, iron supplementation with products other than the investigational drug was prohibited, as were blood transfusion, and erythropoiesis stimulating agents.

### Objective and endpoints

This pooled analysis evaluated the efficacy and safety of FDI compared with IS in a population with IDA after bariatric surgery. The analysis documented all doses of IV iron administered at baseline and at Weeks 1, 2, and 3. All outcomes in the post hoc analysis were assessed at the post-baseline time points that were shared by the two trials (Weeks 1, 2, and 4). Efficacy outcomes included the change in Hb, *s*-ferritin, and TSAT levels from baseline, the proportion of responders (defined as participants with an Hb concentration increase ≥ 2 g/dL from baseline), the time to achieve a treatment response, and the proportion of participants achieving target iron parameters (*s*-ferritin ≥ 100 ng/mL and TSAT of 20–50%). Safety outcomes included the incidence of ADRs (i.e., the proportion of patients with ADRs), the incidence of treatment-emergent serious or severe HSRs, and laboratory assessments, such as the change in serum calcium (*s*-calcium) concentration from baseline, and the incidence of hypophosphatemia (serum phosphate [*s*-phosphate] < 2.0 mg/dL) and severe hypophosphatemia (*s*-phosphate < 1.0 mg/dL). Serious or severe HSRs were defined by a standardized set of Medical Dictionary for Regulatory Activities (MedDRA) terms. The MedDRA terms are listed in the supplementary material of the FERWON-IDA trial publication [44].

### Data analysis sets

Safety analyses were conducted on the safety analysis set (SAS; N = 159), defined as all randomized participants who received at least one dose of the trial medication. Efficacy analyses were conducted on the full analysis set (FAS; N = 159), which included all participants in the SAS who had at least one post-baseline Hb measurement. In this pooled analysis, the SAS and the FAS represented the same population.

### Statistical analyses

Data are presented as mean (standard deviation [SD]) and least squares mean (95% confidence interval) for continuous variables, and as the number and percentage of participants for categorical variables.

Baseline laboratory parameters were compared between the treatment groups using a Wilcoxon rank-sum test. A mixed model for repeated measures with trial, treatment, and day as factors, treatment-by-day and baseline value-by-day interactions, and baseline value as covariate was used to compare the mean changes in Hb, *s-*ferritin, TSAT, and *s*-calcium. The proportion of responders, and participants with *s*-ferritin ≥ 100 ng/mL and TSAT of 20–50%, were compared between the treatment groups using a Fisher’s exact test. Time to treatment response was estimated using a Kaplan–Meier method, and the treatment groups were compared using a log-rank test. The incidences of ADRs, serious or severe HSRs, and hypophosphatemia were compared between the treatment groups using a Fisher’s exact test. All statistical tests were two-tailed with a significance level of 0.05. All analyses were performed using SAS software (version 9.4).

## Results

### Population

This pooled analysis included a total of 159 patients with IDA occurring after bariatric surgery: 93 received FDI and 66 IS. Demographics and baseline laboratory parameters are summarized in Table 2. Caucasian women constituted the majority of the population, and the most common type of bariatric surgery was gastric bypass (>85% of cases). On average, bariatric surgery had been conducted > 8.5 years before the trial. Baseline characteristics were comparable among the treatment groups, although *s-*ferritin and TSAT levels were numerically higher in the FDI group compared with the IS group. *S*-phosphate and *s*-calcium levels were similar in both treatment groups and were within the reference ranges defined by the central laboratories used in the two trials.

**Table 2:** Demographics and baseline laboratory parameters

	FDI (N = 93)	IS (N = 66)
Demographics		
Age (years)	47.6 (10.8)	45.3 (10.2)
Gender, N (%)		
Women	91 (97.8)	64 (97.0)
Men	2 (2.2)	2 (3.0)
Race, N (%)		
White	78 (83.9)	51 (77.3)
Black or African American	13 (14.0)	14 (21.2)
Native Hawaiian or other Pacific Islander	0 (0.0)	1 (1.5)
Other	2 (2.2)	0 (0.0)
Weight (kg)	89.9 (22.6)	89.5 (22.3)
BMI (kg/m^2^)	33.5 (8.0)	33.3 (8.5)
Bariatric surgery, N (%)		
Gastric bypass	80 (86.0)	57 (86.4)
Gastric banding	3 (3.2)	2 (3.0)
Obesity surgery	3 (3.2)	0 (0.0)
Metabolic surgery	3 (3.2)	4 (6.1)
Gastrectomy (sleeve gastrectomy)	4 (4.3)	3 (4.5)
Time since bariatric surgery (years)^a^	9.7 (6.7) (n = 92)	8.6 (7.3) (n = 66)
Laboratory parameters		
Hb (g/dL)	9.3 (1.0)	9.2 (1.3)
*S*-ferritin (ng/mL)	9.3 (14.3)	6.2 (4.0)
TSAT (%)	6.9 (13.2)	5.1 (2.8)
*S*-phosphate (mg/dL)	3.7 (0.5) (n = 89)	3.6 (0.6) (n = 63)
*S*-calcium (mg/dL)	8.9 (0.4) (n = 89)	8.9 (0.4) (n = 63)

Data are presented for the FAS; data presented are mean (SD) unless otherwise stated

^a^Time between the date of surgery (all types of bariatric surgery) and the date of first dose in the trial

BMI, body mass index; FAS, full analysis set; FDI, ferric derisomaltose/iron isomaltoside 1000; Hb, hemoglobin; IS, iron sucrose; N, number of patients; SD, standard deviation; *s*-calcium, serum calcium; *s*-ferritin, serum ferritin; *s*-phosphate, serum phosphate; TSAT, transferrin saturation

### Exposure to iron

The mean (SD) cumulative iron dose was 1199 (± 347) mg with FDI and 937 (± 209) mg with IS during the first 3 weeks of the trials. Of the participants treated with FDI, 73.1% (68/93) received a single administration and 26.9% (25/93) received two administrations. The IS group received between two and seven administrations; the majority of individuals (68.2%; n = 45/66) received five administrations.

### Change in hemoglobin

**Fig. 1:**
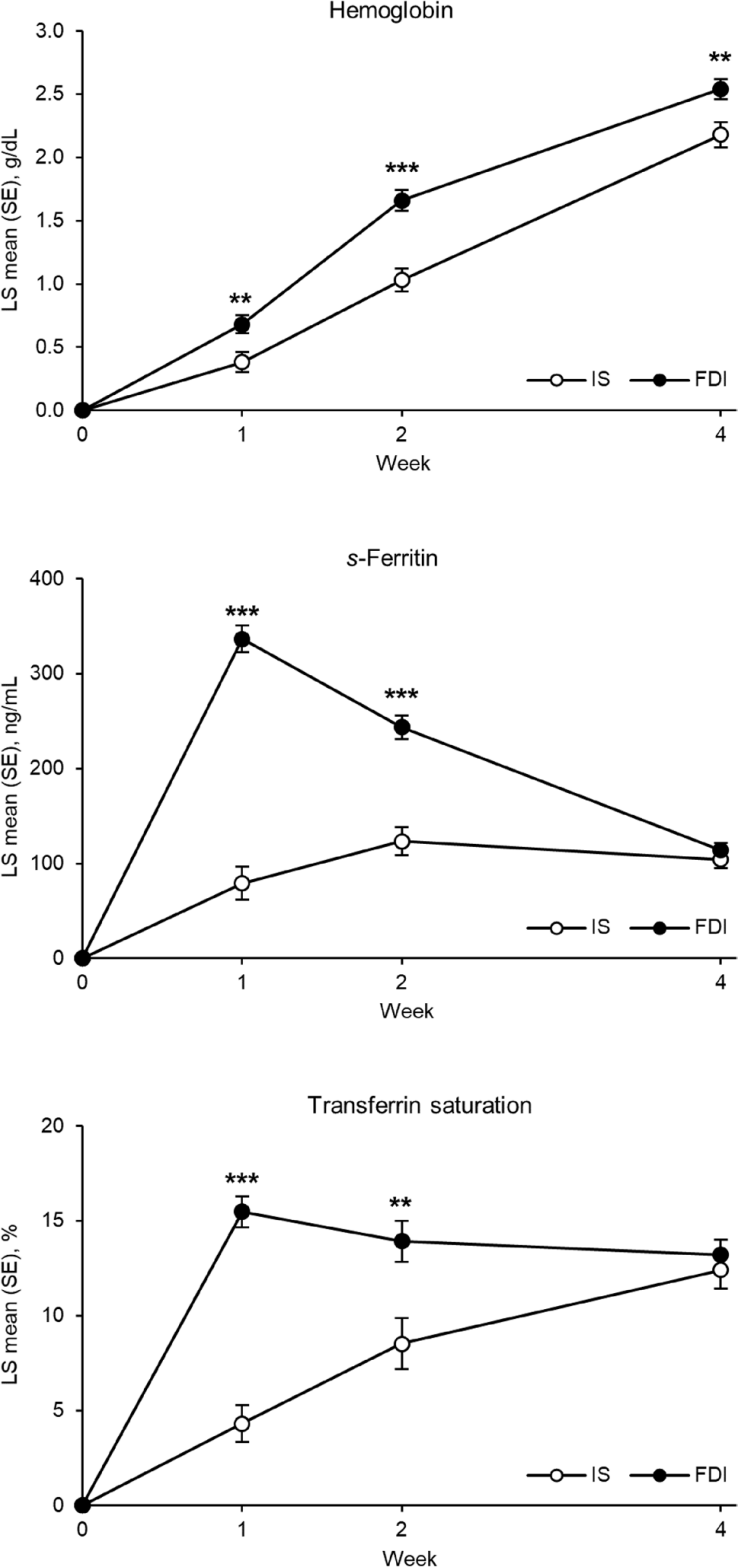
LS mean change in hematological parameters from baseline over 4 weeks. **p < 0.01, ***p < 0.001 versus IS; estimates from mixed model for repeated measures with study, treatment and day as factors, treatment*day and baseline*day interactions, and baseline value as covariate. Data are presented for the FAS. FAS, full analysis set; FDI, ferric derisomaltose; IS, iron sucrose; LS, least squares; SE, standard error

Hb concentration increased more rapidly and to a greater extent from baseline to Week 4 with FDI compared with IS. The increase in Hb was significantly higher with FDI than IS at Weeks 1 (p<0.01), 2 (p<0.0001), and 4 (p<0.01; Fig. 1). The proportion of responders (defined as participants with an Hb concentration increase ≥ 2 g/dL from baseline) was higher with FDI than with IS at Weeks 1, 2, and 4. The difference was statistically significant at Week 2 (p<0.0001; Table 3). The time to Hb response was significantly shorter with FDI versus IS (p < 0.01). The number of participants responding at Weeks 1, 2, and 4 was 5, 29, and 30 with FDI, and 0, 4, and 33 with IS.

**Table 3:** Frequency of responders and participants achieving target iron parameters

	FDI n/N (%)	IS n/N (%)	P-value^a^
Participants with Hb level increase ≥ 2 g/dL from baseline			
Week 1	5/91 (5.5)	0/62 (0.0)	0.0810
Week 2	33/91 (36.3)	4/61 (6.6)	< 0.0001
Week 4	63/91 (69.2)	37/61 (60.7)	0.2989
Participants with *s*-ferritin ≥ 100 ng/mL and TSAT of 20–50%			
Week 1	56/88 (63.6)	3/63 (4.8)	< 0.0001
Week 2	42/91 (46.2)	5/59 (8.5)	< 0.0001
Week 4	26/90 (28.9)	14/60 (23.3)	0.5722

Data are presented for the FAS

^a^FDI versus IS using a Fisher’s exact test

FAS, full analysis set; FDI, ferric derisomaltose/iron isomaltoside 1000; Hb, hemoglobin; IS, iron sucrose; n, number of responders; N, number of patients; *s*-ferritin, serum ferritin; TSAT, transferrin saturation

### Changes in serum ferritin and transferrin saturation

*S*-ferritin concentration and TSAT increased more rapidly and to a greater extent with FDI versus IS at Weeks 1 and 2 (*s*-ferritin, p<0.0001 at both time points; TSAT, p < 0.0001 at Week 1, and p < 0.01 at Week 2), but there were no significant differences between the groups at Week 4 (Fig. 1). At all time points assessed, the proportion of participants achieving target iron parameters (*s*-ferritin ≥ 100 ng/mL and TSAT of 20–50%) was higher in the FDI group, and the difference was statistically significant at Weeks 1 and 2 (p < 0.0001 at both time points; Table 3).

### Adverse drug reactions and hypophosphatemia

The incidence of ADRs was similar, < 20% in the FDI and IS groups, although the number of ADRs was twice as high with IS compared with FDI (Table 4). None of the ADRs were considered serious, and no serious or severe HSRs were reported. The most common ADRs (≥ 3% in any group) included constipation, myalgia, nausea, headache, dysgeusia, fatigue, hyperhidrosis, and vomiting, with no significant differences observed between the FDI and IS treatment groups (Table 4).

**Table 4:** Incidence of ADRs over 4 weeks from first exposure

	FDI (N = 93)		IS (N = 66)		P-value^a^
	N (%)	E	N (%)	E	
ADRs	14 (15.1)	20	12 (18.2)	43	0.6657
Serious ADRs	0 (0.0)	0	0 (0.0)	0	NA
ADRs (MedDRA preferred term) with incidence ≥ 3% in any group					
Constipation	4 (4.3)	4	0 (0.0)	0	0.1420
Myalgia	1 (1.1)	1	2 (3.0)	3	0.5705
Nausea	1 (1.1)	1	3 (4.5)	4	0.3080
Headache	1 (1.1)	1	2 (3.0)	2	0.5705
Dysgeusia	0 (0.0)	0	2 (3.0)	4	0.1708
Fatigue	0 (0.0)	0	2 (3.0)	4	0.1708
Hyperhidrosis	0 (0.0)	0	2 (3.0)	2	0.1708
Vomiting	0 (0.0)	0	2 (3.0)	2	0.1708

Data are presented for the SAS

^a^Number of patients with FDI versus IS using a Fisher’s exact test

ADR, adverse drug reaction; E, number of events, FDI, ferric derisomaltose/iron isomaltoside 1000; IS, iron sucrose; MedDRA, Medical Dictionary for Regulatory Activities; N, number of patients; NA, not applicable; SAS, safety analysis set

The incidence of hypophosphatemia (*s*-phosphate < 2.0 mg/dL) was 0.0% (0/91) in the FDI group and 1.6% (1/63) in the IS group at Week 1, and 3.3% (3/91) in the FDI group and 0.0% (0/59) in the IS group at Week 2. At both time points, the differences between the treatment groups were not statistically significant. At Week 4, there were no cases of hypophosphatemia. None of the participants developed severe hypophosphatemia (*s*-phosphate < 1.0 mg/dL).

### Serum calcium

*S*-calcium was stable across the 4 weeks and remained within reference range in both treatment groups (mean levels remained at approximately 9 mg/dL with FDI and IS); no significant differences were observed between the groups.

## Discussion

In this post hoc analysis of pooled data from the PROVIDE and FERWON-IDA trials, FDI resulted in faster and more pronounced hematological responses compared with IS in the subgroup with IDA following prior bariatric surgery. The time to achieve an Hb increase ≥ 2 g/dL was also significantly shorter with FDI than with IS. These data are consistent with the main analyses of the PROVIDE and FERWON-IDA trials, which included patients with IDA of various etiologies [43, 44].

The opportunity to give higher doses of FDI in fewer administrations, within a shorter time period, compared with IS, possibly accounted for the faster and more pronounced improvements in hematological parameters observed with FDI. At least five infusions of IS would be required to achieve the same dose as a single 1000 mg infusion of FDI. In this analysis, the modal number of infusions was one with FDI and five with IS. Reduced dosing frequency is a key advantage of FDI over IS, which in turn can reduce costs [27, 28].

Another high-dose IV iron product—ferric carboxymaltose (FCM)—has demonstrated effectiveness in bariatric surgery patients with ID, with or without anemia [24, 47]. Although FDI and FCM have not been compared directly in a bariatric surgery population, clinical trials across various therapeutic indications have shown that FDI and FCM have similar efficacy in the treatment of IDA of various etiologies [37, 48, 49].

The population in this analysis reflects the expected characteristics of patients with IDA resulting from bariatric surgery. The majority of patients were women, who are at higher risk of developing IDA than men [1, 6]. In this analysis, > 85% had undergone a gastric bypass procedure—the operation associated with the highest incidence of IDA [5, 6, 10].

In this pooled analysis, FDI and IS were well tolerated with a similar incidence of ADRs (15.1% and 18.2%, respectively), and no serious ADRs were observed. Similar ADR profiles were also reported in the mixed IDA populations in the PROVIDE and FERWON-IDA parent trials [43, 44].

The potential for serious or severe HSRs with IV iron is a concern among medical professionals though, in reality, serious or severe HSRs are rare [32, 50]. In this pooled analysis, no serious or severe HSRs were reported with either formulation. These data are consistent with the current findings in populations with IDA of various etiologies. An analysis of data from high-quality randomized controlled trials found a low incidence of serious or moderate-to-severe HSRs (0.2–1.7%) with newer IV iron formulations, and no statistically significant differences between formulations [32]. Furthermore, a comprehensive meta-analysis of data from trials enrolling more than 8500 patients with IDA of various etiologies confirmed the low incidence of serious or severe HSRs (0.6–1.6%) with modern IV iron formulations [50]. Reports of IV iron-induced HSRs specifically in the bariatric surgery population also show low rates of serious or severe HSRs [24, 47], with no differences from the mixed IDA population.

Hypophosphatemia is a concern with certain IV iron products and can have important clinical consequences, particularly when the hypophosphatemia is severe and persistent [38]. Bariatric surgery patients may be particularly susceptible to hypophosphatemia in the context of IV iron treatment, as they often develop secondary hyperparathyroidism due to vitamin D deficiency and calcium malabsorption, which can lower *s*-phosphate levels [51, 52]. In this analysis of post bariatric surgery patients, rates of hypophosphatemia were low with FDI and IS, and were consistent with the rates observed in the mixed IDA populations in the PROVIDE and FERWON-IDA trials [43, 44]. Importantly, no cases of severe hypophosphatemia were observed.

The present analysis has limitations. The analysis was conducted post hoc, which precludes robust conclusions. However, the results are consistent with the preponderance of published evidence demonstrating the efficacy of IV iron in the treatment of ID/IDA following bariatric surgery [24, 47].

Another limitation is the short treatment period used in this analysis. Bariatric surgery patients are highly susceptible to ID and, therefore, may require re-treatment with IV iron. Consequently, it is important to understand the long-term efficacy and safety of IV iron in this population. Although this analysis did not explore the long-term effects of FDI and IS, a 6-month extension study (FERWON-EXT) found that re-dosing patients with FDI resulted in rapid improvements in Hb levels and low rates of ADRs [53]. FERWON-EXT enrolled patients from the PROVIDE, FERWON-IDA, and FERWON-NEPHRO trials [53], and included individuals with IDA resulting from prior bariatric surgery.

In conclusion, in patients with IDA following bariatric surgery, FDI treatment was delivered in fewer visits and was associated with a faster and more pronounced hematological response than IS. FDI was well tolerated with a similar incidence of ADRs to IS and no cases of severe hypophosphatemia, or serious or severe HSRs.
